# Temporomandibular Disorders: Current and Future Concepts in Diagnosis and Management

**DOI:** 10.3390/medicina59020223

**Published:** 2023-01-24

**Authors:** Martina Ferrillo

**Affiliations:** Dentistry Unit, Department of Health Sciences, University of Catanzaro “Magna Graecia”, 88100 Catanzaro, Italy; martina.ferrillo@unicz.it; Tel.: +39-09617-12819

Temporomandibular disorders (TMD) are musculoskeletal and/or neuromuscular conditions relating to muscles, joints, and the associated structures of the stomatognathic system [1]. To allow clinicians to adopt a common *iter* for the diagnosis of TMD patients, the diagnostic criteria for TMD (DC/TMD) were established in 2014 [1].

Based on patient signs and symptoms, the DC/TMD defines two axes: (i) Axis I, categorizing the TMD into muscular TMD (Group I) and arthrogenous TMD (Group II and III); (ii) Axis II, assessing the disability due to the TMD pain through the evaluation of behavioral and psychological status [1].

An overall prevalence of TMD patients of around 30% has been recently reported, consisting of around 45% for group I and 70% for groups II and III (taking into account the overlapping conditions) [2].

The etiology of TMD has been accepted as multifactorial, and the suggested risk factors can include excessive muscle tension, grinding, clenching as a parafunctional activity, repetitive trauma to the joint, and bruxism [3]. Furthermore, psychological and emotional distress should be considered when managing chronic pain, considering that anxiety and depression might lead to the development and/or exacerbation of pain [2].

Myofascial pain prevalence is higher in primary pain conditions connected to the central nervous system, including headaches and other chronic conditions, most likely due to central sensitization [2,4]. Indeed, in addition to increased temporal pain, central sensitization might induce hypersensitivity, increased pain sensation, or allodynia, defined as pain deriving from non-noxious stimuli [2].

It has been shown in the scientific literature that the most common signs and symptoms of TMD patients are pain and the articular limitation of jaw motion; these might even induce disability during activities of daily living (e.g., talking, swallowing, etc.) with a consequent reduction in health-related quality of life [5].

Furthermore, it has to be noted that the recent COVID-19 epidemic might lead to a higher frequency of TMD symptoms, interacting with the psychological and emotional status of the population [6]. Indeed, it is well known that COVID-19 can induce increased disability [7] and higher psychological distress that might become a potential amplifier of central pain sensitization in TMD patients [2].

The diagnosis is commonly performed according to DC/TMD and through cone beam computer tomography and magnetic resonance imaging exams, commonly required for the diagnosis of temporomandibular joint (TMJ) disorders [8]. The MRI is accepted as the reference standard for evaluating inflammatory conditions and soft tissue areas, including muscles, ligaments, and the cartilaginous disc of TMJ; on the other hand, CBCT is recommended for evaluating hard skeletal and dental tissues [8].

Concerning the treatment of TMD patients, the main objectives that should be focused on are: (i) reducing TMJ pain; (ii) reducing masticatory muscle pain; (iii) improving TMJ function; and (iv) avoiding further TMJ impairments [9,10]. In this scenario, the conservative approach might be considered as first-line therapy for TMD patients, including different interventions, such as: (i) behavioral therapy; (ii) physiotherapy; (iii) transcutaneous electrical nerve stimulation; (iv) laser therapy; (v) extracorporeal shockwaves therapy; and (vi) oxygen–ozone therapy [9,10,11,12,13,14].

Lastly, telemedicine might allow us to monitor TMD patients over time, particularly during the COVID-19 pandemic, providing adequate feedback [6,15]. In this scenario, “teledentistry” can offer the possibility to continue the dental practice using this novel intervention in parallel to conventional therapeutic approaches in dentistry and oral rehabilitation [15,16].

Therefore, multidisciplinary intervention is required for managing TMD pain, taking into consideration an appropriate diagnosis and all the interdisciplinary therapeutic approaches concerning the TMJs and the neuromuscular structures of the masticatory system. Further evidence is still needed to better characterize the physiopathology, diagnosis, and treatment of TMD patients.
